# Smallholder perceptions of land restoration activities: rewetting tropical peatland oil palm areas in Sumatra, Indonesia

**DOI:** 10.1007/s10113-020-01737-z

**Published:** 2020-12-19

**Authors:** Caroline Ward, Lindsay C. Stringer, Eleanor Warren-Thomas, Fahmuddin Agus, Merry Crowson, Keith Hamer, Bambang Hariyadi, Winda D. Kartika, Jennifer Lucey, Colin McClean, Neneng L. Nurida, Nathalie Petorelli, Etty Pratiwi, Aasmadi Saad, Ririn Andriyani, Tantria Ariani, Heni Sriwahyuni, Jane K. Hill

**Affiliations:** 1 Sustainability Research Institute, University of Leeds, Leeds, UK; 2 Leverhulme Centre for Anthropocene Biodiversity, University of York, York, UK; 3 Environment Department, University of York, York, UK; 4 Department of Biology, University of York, York, UK; 5 School of Natural Sciences, Bangor University, York, UK; 6 Indonesia Soil Research Institute, Indonesia Center for Agricultural Land Resources Research and Development, Bogor, Indonesia; 7 Institute of Zoology, Zoological Society of London, London, UK; 8 School of Biology, University of Leeds, Leeds, UK; 9 Biology Education Program, Faculty of Education and Teacher Training, Jambi University, Jambi, Indonesia; 10 Department of Zoology, University of Oxford, Oxford, UK; 11 Soil Science Division, Faculty of Agriculture, Jambi University, Jambi, Indonesia

**Keywords:** Conservation social science, Environmental social science, Perceptions, Questionnaires, Interviews

## Abstract

The Indonesian government committed to restoring over 2 million ha of degraded peatland by the end of 2020, mainly to reduce peat fires and greenhouse gas emissions. Although it is unlikely the government will meet this target, restoration projects are still underway. One restoration strategy involves blocking peatland drainage canals, but the consequences of this for smallholder farmers whose livelihoods are dependent on agriculture are unclear. This paper investigates perceived impacts of canal blocks on smallholder farmers and identifies factors that affect their willingness to accept canal blocks on their land. We use data from 181 household questionnaires collected in 2018 across three villages in Jambi province, Sumatra. We found that the majority of respondents would accept canal blocks on their farms, perceiving that the blocks would have no impact on yields or farm access, and would decrease fire risk. Respondents who would not accept blocks on their farms were more likely to use canals to access their farms and perceive that canal blocks would decrease yields. The majority of farmers unwilling to accept canal blocks did not change their mind when provided with an option of a block that would allow boat travel. Our results improve understanding of why some smallholders may be unwilling to engage with peatland restoration. Further research is needed to understand the impact of canal blocks on smallholders’ yields. Engaging with stakeholders from the outset to understand farmers’ concerns, and perceptions is key if the government is to succeed in meeting its peatland restoration target and to ensure that the costs and benefits of restoration are evenly shared between local stakeholders and other actors.

**Supplementary Information:**

The online version contains supplementary material available at 10.1007/s10113-020-01737-z.

## Introduction

Tropical peatlands play important roles as global carbon sinks (Jauhiainen et al. 2016) and forest habitats for endangered species (Posa et al. 2011) and provide ecosystem services for local people, including provisioning services such as food, materials and medicinal plants (Kimmel and Mander 2010). Once considered marginal areas, peatlands are increasingly exploited for agriculture, especially oil palm and wood fibre cultivation by both large-scale industrial plantations and smallholder farmers (Miettinen et al. 2012; Wijedasa et al. 2017). This requires drainage and vegetation clearance, leading to peatland degradation (Green and Page 2017). Peatlands are commonly drained via the construction of canals (from small hand-dug canals of 1 m width, to industrial drainage canals >30 m width), which become important for accessing farm land and transporting crops and materials (Page et al. 2009; Dohong et al. 2018; Hansson and Dargusch 2018). Once peatlands have been cleared and drained (‘degraded’), the water table is lowered away from the ground surface, enabling crops which would not survive in flooded land to be planted. However, a range of issues can ensue, including subsidence, carbon emissions (tropical peatlands sequester and store carbon above and below ground) and biodiversity loss (Miettinen et al. 2012; Jauhiainen et al. 2016; Page and Baird 2016; Green and Page 2017; Wildayana et al. 2018). Drained peatlands are also susceptible to fires, which have further negative consequences for greenhouse and toxic gas emissions, lead to economic damage, negative livelihood impacts, biodiversity loss and significant public health burdens (Marlier et al. 2015; Koplitz et al. 2016; Page and Baird 2016; Sze et al. 2018).

**Fig. 1:**
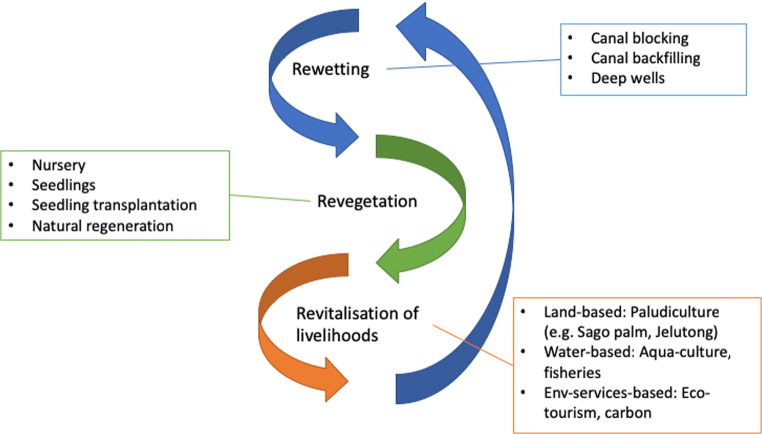
Indonesia’s Peatland Restoration Agency (Badan Restorasi Gambut, BRG) three Rs of peatland restoration (adapted from Dohong 2017)

Peatland restoration, i.e. the process of assisting the recovery of peatland that has been degraded or damaged towards an agreed baseline condition (Ritzema et al. 2014; Graham et al. 2017; Dohong et al. 2018) is a relatively new initiative in tropical areas (Page et al. 2009). A range of management interventions have sought to restore degraded peatlands (Dohong 2017; Graham et al. 2017; Jefferson et al. 2020). Indonesia provides a useful case in which to investigate restoration interventions, because the national government pledged to restore more than 2 million ha of peatland by the end of 2020 (Wardhana 2016) across both plantation concessions and smallholder land, chiefly for the purposes of reducing peat fires and greenhouse gas emissions (Wardhana 2016; Evers et al. 2017). This action was largely motivated by the extreme fire event of 2015 which had severe national and regional impacts. Haze from the 2015 fires extended to Singapore, Malaysia and Thailand leading to respiratory illnesses that contributed to an estimated 100,000 deaths within southeast Asia (Koplitz et al. 2016) and economic losses of US$16.1 billion (World Bank, 2015) in Indonesia alone. To ensure the restoration pledge is met, the Peatland Restoration Agency (Badan Restorasi Gambut, BRG) was established in 2016. BRG’s approach revolves around the ‘three Rs’: rewetting, revegetation and revitalisation of livelihoods (Fig. 1). Concession-holders are responsible for restoration in plantation areas (Dohong 2017). In this paper, we focus on smallholder land. Whilst relatively small-scale or trial peatland restoration projects in Indonesia had been established by NGOs prior to the government’s restoration pledge, e.g. the Mega Rice project in Kalimantan (Page et al. 2009; Schaafsma et al. 2017), these were insufficiently widespread to be able to prevent nationally and regionally significant economic impacts from the 2015 fires and, in some cases, had more negative than positive impacts (Dohong and Lilia 2008; Jaenicke et al. 2011; Graham et al. 2017).

By the end of 2019, it was reported that BRG had restored less than 780,000 ha, although there is little information available on overall progress towards the target, and criticisms have been raised over the maintenance of restoration infrastructure, particularly canal blocks and wells (Jong 2020a; Ward et al. 2020). Peatland fires decreased from 2015 to 2018, but increased again in 2019 (Haniy et al. 2019; Reuters 2019), and there are concerns that a focus on COVID-19 in 2020 may impact funds and resources, leading to increased fires again (Jong 2020b). Journalists have also reported that BRG may be dissolved and merged with other government departments at the end of 2020 (Ibnu 2020). Despite the precarity of BRG’s position, peatland restoration is likely to remain a focus for Indonesia given the issues with fire and commitments to reducing carbon emissions.

In this paper, we focus on rewetting, which involves constructing canal blocks (dams) or backfilling drainage canals, in order to prevent further drainage and raise the water table. Despite the central role of rewetting within BRG’s three-Rs approach, the consequences for smallholder farmers, whose livelihoods depend on agriculture and whose land sits within the canal block areas, demands further urgent investigation. In this paper, we explore smallholder farmer perceptions of peatland rewetting in order to help address this current gap in understanding. Researchers, NGO and government guidelines suggest that rewetting should take place in conjunction with other interventions, such as paludiculture (cultivation of crops adapted to wet/peat soil), other livelihood projects and revegetation (replanting of native peat species) (Fig. 1; Page et al. 2009; Dohong 2017; Graham et al. 2017). Several different canal block designs and construction materials have been trialled depending on whether the peatland is currently under human use, the available materials and the size of drainage canals (Dohong 2017). We focus on canal blocking as it has been identified as the most important intervention for successful restoration and has had the greatest focus in terms of actions taken, and it is likely to have an impact relatively quickly (compared to revegetation; Dohong 2017; Graham et al. 2017; Ward et al. 2020). For production areas (i.e. any area being used to grow any commercial crop) on peat soils, the government issued a decree in 2014 that the water table should be maintained at 0.4 m or higher, relative to the peat surface (Dohong 2017). There nevertheless appears to be little scientific evidence behind this decision (Page et al. 2009; Wardhana 2016; Dohong et al. 2018; Sabiham et al. 2018). Existing studies on the efficacy of canal blocks are somewhat limited and have tended to focus on the biophysical aspects of rewetting. For example, research has shown that canal blocks can raise water table depth, but that they can also be susceptible to erosion or damage from extreme weather and do not seem able to return water table depths to expected natural levels (Ritzema et al. 2014; Dohong et al. 2018).

Although agriculture on peatland is also undertaken by large companies, we focus on canal blocks on land used by smallholder farmers in this study. ‘Smallholder’ farmers can be a difficult term to define as farm sizes and types differ between countries (Stringer et al. 2020). Even within countries, smallholders are a heterogenous group (Jelsma et al. 2017). In this research, we follow the RSPO (2020) definition of smallholders: ‘… farmers who grow oil palm, alongside with subsistence crops, where the family provides the majority of labour and the farm provides the principal source of income, and the planted oil palm area is less than 50 ha’. Peatland is classified as marginally suitable for agriculture, due to its waterlogged, high acidity and poor nutrient soil content, and needs high inputs to increase productivity (Hergoulac’h et al. 2017). Yet many household livelihoods globally rely on peatland areas for largely market-based agricultural activities (Luskin et al. 2014; Wildayana 2017). In Indonesia, smallholder farmers were encouraged to plant oil palm by government-backed contracts in the 1970s, and this slowly moved into contracts with oil palm mills and cultivation of oil palm by independent farmers who do not have a contract with a specific mill (McCarthy et al. 2012; Jelsma et al. 2017). Globally, smallholders contribute 40% of the global palm oil supply (Euler et al. 2017; Kubitza et al. 2018), and in Indonesia, smallholders were responsible for 60% of peatland conversion to agriculture during the period of 1990–2010 (Wijedasa et al. 2018). Such conversion has significantly improved the livelihoods of many rural households. In Sumatra, studies have not only shown that the uptake of smallholder oil palm has improved household living standards and nutrition, but has also widened inequalities as wealthier households have had the largest economic gains (Rist et al. 2010; Euler et al. 2017; Kubitza et al. 2018). Although there have been some studies looking at institutional-level social and economic dimensions of peatland rewetting, particularly focussing on fire management (e.g. Carmenta et al. 2017; Sze et al. 2018; Jefferson et al. 2020), the smallholder farmer perspective remains under-researched. Despite the lack of attention, the smallholder perspective is important to consider given that effective canal blocks require the support of stakeholders to maintain them, especially when canals have multiple uses, not only for drainage but also for transport. Canal blocks may also have negative impacts on smallholder farmers. Raising the water level in agricultural areas may reduce yields of certain crops or impede harvests, leading to detrimental impacts on local livelihoods despite the other potential benefits it offers (e.g. cleaner water, reduced fire risk (Bryan, 2014) and reduced CO2 emissions (Jauhiainen et al. 2016)). Monitoring of restoration interventions is also more difficult in smallholder farms compared with large-scale plantations. Moreover, decisions about which sites to restore need to be compatible with systems of local governance, property rights and devolved administrations (Carmenta et al. 2017). This suggests local stakeholder involvement in restoration decisions is necessary and is supported by findings from a recent study that found researchers, government officials and NGOs all considered local involvement to be crucial to peatland restoration success in Indonesia (Ward et al. 2020).

Understanding stakeholder perceptions of environmental management interventions is critical to improve their design and on-the-ground implementation, for both instrumental and ethical reasons (Bennett 2016; Carmenta et al. 2017). It is also fundamental to ensuring legitimacy and buy-in, enabling transparent boundary management and incorporating knowledge and interests across scales (de Vente et al. 2016; Sterling et al. 2017; Stringer et al. 2018). In the case of canal blocking in tropical peatland areas, there is limited published research of the impacts on and perceptions of smallholder farmers living in or near locations where canal blocks have been constructed. A few studies and reports mention issues with farmers being unsupportive of restoration efforts, with some cases of canal blocks being destroyed (e.g. Dohong and Lilia 2008; Dohong et al. 2018). If restoration and rewetting activities are to be successful, then further research is needed to understand why smallholder farmers may have negative perceptions of canal blocks and to create solutions that can continue restoration efforts without negatively impacting local stakeholders. This paper helps to fill this research gap by focussing on smallholder perceptions of canal blocks, identifying the factors that affect the acceptance of a canal block being built on smallholder farms. We focus on Indonesia as a study country, with field sites in Sumatra (see ‘Methodology’). We explore (1) whether smallholder farmers would agree to a scenario of canal blocks being built on their farms, why and what factors influence this decision; (2) how smallholders perceive canal blocks will impact their yields, farm access and fire risk; and (3) for smallholders not willing to have canal blocks built on their farms, whether they would accept different canal block designs.

We consider perceptions, rather than solely focusing on objective measurements or indicators of the impacts of installing canal blocks. Perceptions are important in understanding and influencing human behaviours (Ajzen 1991), enlisting stakeholders’ support (Gurney et al. 2015) and minimising negative impacts of environmental management interventions. Yet, perceptions are frequently criticised as not being reliable evidence, as they are subjective, may not accurately represent outcome variables, can be purposefully inaccurate and cannot be used to determine causality (Bennett 2016). Perceptions are highly mediated by past experiences and personal motivations, meaning that they can be highly heterogeneous within geographical, livelihood or socio-economic groups, but this is also where their strength as a form of evidence lies. Perceptions can be used to provide insight and are particularly useful in understanding the legitimacy and acceptability of management actions (Cinner and Pollnac 2004; Martin et al. 2014; Bennett and Dearden 2014; Carmenta et al. 2017). Therefore, perceptions can provide vital insights into improving understanding the subjective ‘how and why’ of local smallholders’ experiences of environmental management interventions such as canal blocks.

## Methods

### Study area

This study was jointly undertaken by various UK and Indonesian institutions, focussing on the area of peatland surrounding Sungai Buluh Peat Protection Forest (Hutan Lindung Gambut, HLG), in the lowlands of Jambi province, Sumatra. We chose Sumatra as there has been less research effort on peatlands here, compared with Kalimantan. However, we believe that some of our findings will be applicable to other peatland areas within Indonesia. Jambi province has been identified as a fire hotspot, with fires occurring mainly in degraded peatland, and fire risk heightened in El Niño years (Prasetyo et al. 2016; Miettinen et al. 2017). BRG has committed to restoring 151,663 ha of peatland in Jambi, and a number of peatland restoration projects have already begun (Dohong 2017).

**Fig. 2:**
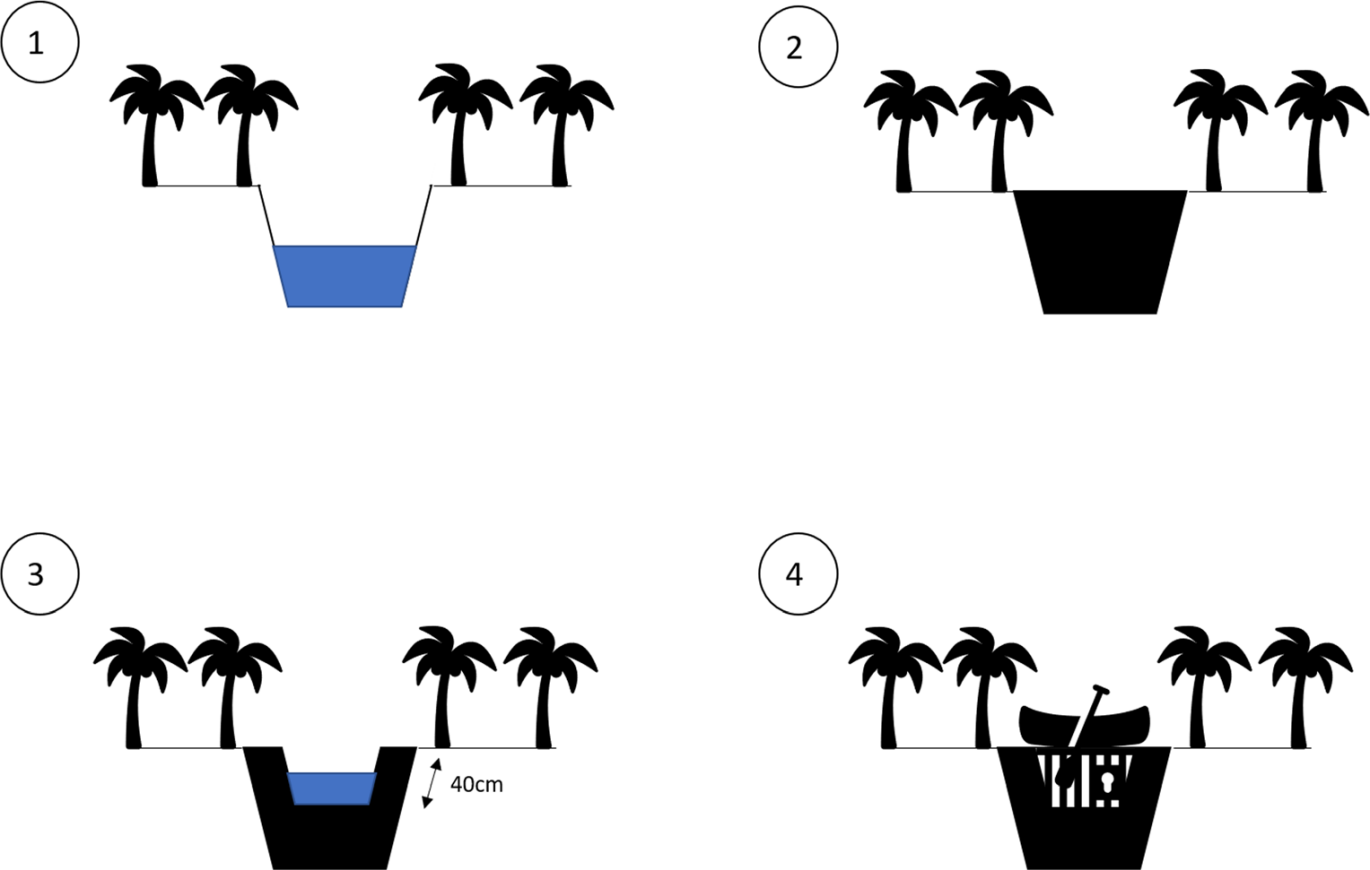
Canal block types: (1) Drainage canal within oil palm farm; (2) full block (construction materials vary) where water is unable to drain at all and canal cannot be used for boat transport (this block type is not usually used in agricultural areas); (3) 40-cm block where the canal is narrowed but leaves a spillway for excess water to drain out and maintaining the water level at 40 cm below ground level (canal cannot be used for boat transport); (4) canal block with gates which can be opened to control water levels and allow boats to pass through canals (in all canal blocks water is still able to drain through lateral flow in the peat soil matrix)

Jambi has been a hotspot of recent oil palm expansion (Krishna et al. 2017), and official statistics show that around 200,000 households (22.9% of households in Jambi) are engaged with growing oil palm (Badan Pusat Statistik 2018). Sungai Buluh Peat Protection Forest is secondary peat swamp forest, having been selectively logged in the past. It is surrounded by agricultural fields and plantations (Crowson et al. 2019). Jambi province has mixed ethnicities with large numbers of people moving to the area during transmigration programmes since 1980, meaning that although the largest group are the indigenous Malays, the second largest constitute Javanese immigrants (Luskin et al. 2014). We included a focus on ethnicity as peatlands are not present on all Indonesian islands, and cultural practices including farming methods differ between islands, so this may affect farmer perceptions. Although we had originally hoped to look at a wider range of restoration interventions, we found that canal blocks were the most frequently implemented intervention in our study area. Livelihood projects (including paludiculture and cattle farming) and revegetation, which in the literature are often described as being implemented parallel to canal blocking, were only present as small trials, and few people had heard about them. We therefore focussed on canal blocks. In our study area, three different types of canal block were observed (Fig. 2): the 40-cm block, where construction of the dam kept the water level at a maximum of 40 cm below the surface, and the rest of the water was able to drain away; full blocks, which prevented any water from continuing to drain; and blocks with gates, where the water level could be managed by farmers and people were still able to use boats on the canals. As the 40-cm block and blocks with gates were the most frequently observed, and according to BRG, are most appropriate for peat cultivation areas (Dohong 2017; Dohong et al. 2018), we chose to focus our data collection on these two types of canal blocks.

### Sampling strategy

We focussed on three villages surrounding the Sungai Buluh Peat Protection Forest. Villages were selected based on willingness to participate, differing numbers and types of canal blocks constructed and comparable livelihood portfolios (i.e. the majority of households in all villages were oil palm farmers). None of these villages had been directly impacted by the 2015 fires, but other areas nearby had experienced fires during the 2015 fire season. We were unable to access accurate, up-to-date population data for the villages, but through conversations with village officials, our sampling strategy aimed to reflect the different sizes of each village, different ethnicities and differing previous experiences of canal blocks. We aimed to obtain a representative sample of smallholders in areas with pre-existing canal blocks and areas without canal blocks. As we were unable to access information on when and where canal blocks had been built and farmers did not necessarily live on or close to their farms, these areas were identified through discussions with village heads and other key stakeholders, such as leaders of farmer groups and other associations. Once areas with canal blocks and without canal blocks in each village had been identified, households were randomly selected and a total of 181 questionnaires were completed.

### Questionnaire

Data collection was via questionnaires with household heads, administered during July–September 2018 (dry season in Sumatra, during a low fire year). Questionnaires were split into four sections: socio-economic information, farm and other livelihood activities, canal block scenarios and previous experience of canal blocks and fire (Online Resource 3). Each canal block scenario included a description and photos of the type of canal block, how it would change water levels (Suryadiputra et al. 2005; Dohong 2017; Dohong et al. 2018) and whether farms would still be able to travel via boat on the canals. The first canal block scenario described a 40-cm block (Online Resource 3). If respondents refused this block, then they were offered a second scenario, which described the block with a gate. This approach meant that we were not asking respondents for their preferred canal block type, but exploring whether the canal block in the second scenario could alleviate the concerns of those respondents who refused the block in the first scenario. This is useful, as BRG publications suggest that 40-cm blocks are likely to be the default as they are cheaper to install and require less maintenance, and there is no responsibility for water management, unlike blocks with gates where someone has to be in charge of when the gates are opened and closed, potentially leading to conflict (Suryadiputra et al. 2005; Dohong 2017; Dohong et al. 2018). After the descriptions, respondents were asked whether they would accept the canal block being built on their land, why and what impact they thought it would have on their crop yield, farm access and fire risk. We also collected data on previous fire experience, current canal use and method of transport used to access farm and harvest crops. A mixture of open-ended and closed questions were used, enabling collection of qualitative and quantitative data, ensuring both depth and breadth of information (Bamberger et al. 2010; Cresswell and Plano Clark 2011) to understand how smallholder farmers perceive canal blocking to impact upon their livelihoods. This combination of methods has been widely used to explore livelihoods and perceptions of environmental restoration (White 2002).

Questionnaire design was informed by discussions with key stakeholders (village officials, farmer groups and BRG members) in April 2018. The questionnaire was written in English and then translated to Indonesian. Questionnaires were administered by 3 Indonesian research assistants from the University of Jambi. Questionnaires were simplified and refined after piloting in July (*n* = 12 for the pilot) which suggested that some questions were too complex. Pilot data was not included in the final sample. Methods were approved by the University of Leeds Ethics Committee before data collection and research approval was given by the Indonesian government (199/SIP/FRP/E5/Dit.KI/VII/2018).

### Data analysis

To assess which factors had the greatest impact on whether smallholders would accept a canal block built in their farm, we used a generalised linear model (GLM), with canal block acceptance as the binomial response variable. We included perceived impacts on yield, farm access, fire risk and a range of socio-economic variables. See Online Resources 1 and 2 for a detailed summary of all the variables included in our model. We assessed the full model for the significance of individual variables and then ran a stepwise selection based on Akaike Information Criteria (AIC) to find the most parsimonious model (Burnham and Anderson 2004). Before carrying out the GLM regression we checked for collinearity by calculating variance inflation factors. All quantitative data analysis was carried out using R (R Core Team 2013).

Qualitative questionnaire responses were analysed using NVIVO software through reading, coding, comparison with quantitative data and recoding (Newing et al. 2011; Sutherland et al. 2018). For qualitative data, thematic analysis enabled categories to be developed for each question, assisting understanding of both the range of answers given and which were the most frequent. This took several rounds of refining categories. No conflicts were found between the findings from qualitative and quantitative data. Qualitative data are used throughout to support or further explain quantitative results.

## Results

### Data summary

As expected for the area, the majority (79.0%) of respondents farmed oil palm as their primary source of income and tended to focus on one or two income-generating activities (Tables 1 and 2). Some (21.0%) oil palm farmers also grew areca nut or coconut alongside, but earned the majority of their income from oil palm. Ethnicities in the villages varied, including people originating from Java, South Sulawesi and different areas in Sumatra. Monthly incomes were highly variable between households, ranging from Rp0.01–100 million per month.

**Table 1:** Summary of household socioeconomic statistics (numerical variables)

Numerical variables	Mean	Standard deviation
Age (years)	42.2	12
Household size (number of people)	4.2	1.3
Income (million rupiah per month)	2.7	1.56
Number of income-generating activities	1.6	0.59

**Table 2:** Summary of household socioeconomic statistics (categorical variables)

Categorical variables	Summary
Village	Village 1: 44.2% Village 2: 22.7% Village 3: 33.1%
Education	None: 8.8% Elementary: 58.6% High School: 20.4% Vocational: 9.4% University: 2.8%
Ethnicity (region respondent was born in)	Born in village: 33.7% Other areas in Sumatra: 26.5% Java: 35.9% Sulawesi: 3.9%
Main income activity	Oil palm: 79.0% Areca nut: 11.0% Coconut: 2.2% Other: 7.7%

### Canal use

The 46.3% of respondents, who stated that they have used the canals within the last year, did so for farm access, drainage and irrigation and to prevent flooding (Online Resource 4). Respondents who defined oil palm as their primary of income were most likely to be using canals, but this was not significantly higher than for households with other income generating activities.

### Previous canal block experience

A total 19.9% of respondents already had canal blocks on their farms, built during the period of 2000–2018 and with a median construction year of 2016. The majority of these were 40-cm blocks (66.7%; see Fig. 2 for overview of canal block types), followed by full blocks (22.2%) and blocks with gates (8.3%), built to rewet or prevent water from draining from their farms (40.5%). Other reasons for canal blocks being built included fire prevention (16.2%), improving irrigation (13.5%) and flood prevention (5.4%). Nearly a quarter of respondents with a canal block on their farm did not know why it had been built. Most canal blocks had been built by the government (55.8%), with smaller numbers constructed by villagers, farmers and plantation companies. A total of 48.6% of respondents felt that their views had not been listened to regarding building the canal block, giving concerns about water levels in wet season and farm access: ‘[I didn’t want the canal block] because I thought it would disturb transportation’ (PR38); ‘I didn’t agree but they built it anyway’ (PL68); ‘I did not want it and now in dry season it is very dry and wet season it floods’ (PR28). However, the majority of respondents also stated that there had been no noticeable impact from canal blocks (61.3%). Some noted difficulty in accessing their farms (12.9%) and lower crop yields (9.7%). No respondents reported positive impacts on yield or farm access. There were no differences in socio-economic variables between respondents with and without canal blocks.

### Canal block scenario 1

**Fig. 3:**
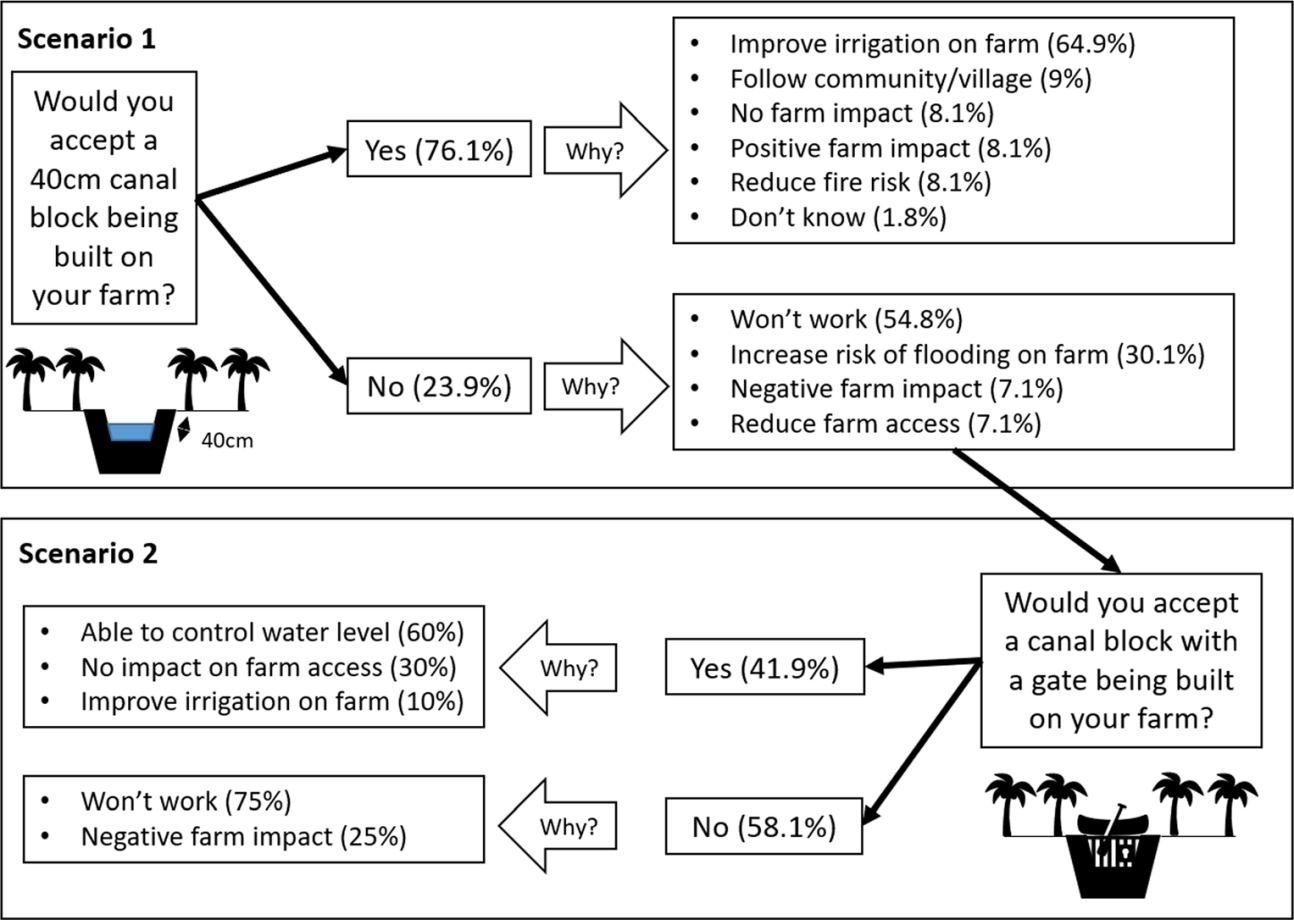
Responses to canal block scenarios and reasons given

The majority (76.1%) of respondents agreed to the scenario of a 40-cm canal block being built on their farm, with the majority of those (64.9%) considering it would improve irrigation on farm. Of the respondents who did not agree to a canal block on their farms, most stated that the canal blocks would not work (54.8%) and felt that the canal water level was also being controlled by tidal changes (see Fig. 3 and Table 3 for other reasons and example quotes).

**Table 3:** Example quotes from the first (40 cm) canal block scenario (with respondent codes denoted in brackets)

Willing to accept canal block	Reason category	Example quotes
Yes	Improve irrigation on farm	‘It will help with irrigation because oil palm needs a lot of water’ (PR26) ‘To help with irrigation and stop the farm from drying out in dry season’ (PL56)
	Follow community	‘As long as it is achieved from discussions with the community’(PL31) ‘I agree with the other people in the village who say canal blocks are good’ (PR36)
	No farm impact	‘It would not matter anyway because we are connected to the [plantation company] canals anyway so we are already affected by their canal blocks’ (PR37) ‘It will not have much impact on the farm or the harvest’ (PL07)
	Positive farm impact	‘It would be good for the oil palm plants’ (PL24) ‘It will improve the harvest’ (M23)
	Reduce fire risk	‘It will prevent burning’ (M53) ‘To reduce the fire risk on the peatland’ (PL43)
No	Will not work	‘It would have no effect because the village is affected by the tide’ (M18) ‘There would be no effect from building it’ (PL23)
	Increase risk of flooding	‘I would be worried that the farm would flood in the rainy season’ (PL25) ‘It would be bad for the oil palm because it will always be wet’ (PR09)
	Negative farm impact	‘It will be bad for the oil palm and the harvest’ (PL16) ‘My farm already has a canal block from [plantation company] and it has a bad impact’ PR40
	Reduce farm access	‘We use the canal for transporting oil palm fruit’ (PL21) ‘It will be bad for accessing farm in wet season’ (M03)

**Fig. 4:**
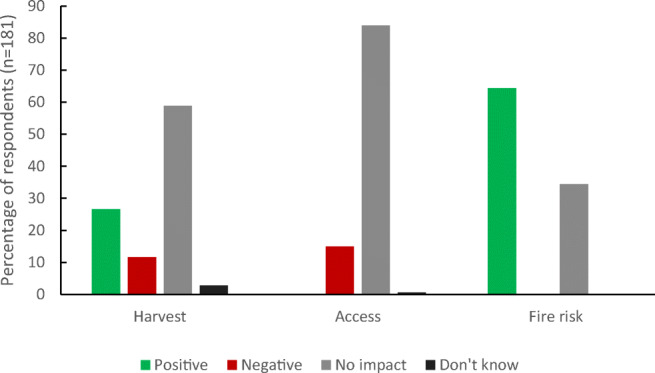
Perceived impacts of 40-cm canal blocks on yields, farm access and fire risk

The majority of respondents perceived that the 40-cm canal block would have no impact on their harvests (58.9%) or farm access (84.4%) and would decrease the risk of fire on their farms (65.2%; Fig. 4). Respondents were divided over whether canal blocks would stop farms from drying out in the dry season or increase the risk of flooding in the wet season (Table 3). A small minority of our respondents (12.4%) relied on boats to access their farms, with the majority accessing their farms by motorbike (59.9%) or walking (26.6%). This finding explains why so few were concerned about impact on farm access.

Results from the binomial GLM show that the two most significant factors in predicting whether a farmer would accept a canal block being built on their farm were perceived impact on harvest and fire risk. Respondents who perceived that the canal block would decrease their harvests were significantly less likely to agree to the canal block (Table 4). This supports the qualitative data explored above, where responses varied between stating that the canal blocks would stop farms from drying out in the dry season and others who thought that canal blocks would increase the risk of flooding in the wet season (see Table 3).

Respondents who perceived that canal blocks would have no impact on fire risk were also significantly less likely to agree to the canal block. Village, ethnicity and farm access were also significant predictors of unacceptance, albeit to a lesser extent. Respondents from village 2 were less likely to agree to canal blocks. Respondents who accessed their farms by walking during wet seasons or those of Sumatran ethnicity were more likely to agree to the canal block.

**Table 4:** Results of the generalised linear model with 40-cm canal block acceptance as the binomial response variable, i.e. a positive value indicates the predictor value increases the likelihood of canal block acceptance. The most significant predictors of canal block acceptance were perceived impacts on harvest and fire risk. Respondents who perceived that canal blocks would decrease their yields and have no impact on fire risk were significantly less likely to agree to the 40 cm canal block scenario

Predictor variables	Estimate	Standard Error	*P* value
(Intercept)	2.303	1.777	0.195
Village 1 (= 1)	− 1.067	0.801	0.183
Village 2 (= 1)	− 3.344	1.078	0.002**
Ethnicity: Java (= 1)	− 0.086	0.683	0.900
Ethnicity: South Sulawesi (= 1)	− 2.117	1.471	0.150
Ethnicity: Sumatra (= 1)	2.269	1.151	0.048*
Age (years)	− 0.025	0.023	0.271
Household size (number of people)	− 0.184	0.221	0.406
Income (million rupiah per month)	0.297	0.272	0.274
Number of income activities	0.362	0.434	0.404
Wet season farm access: motorbike (= 1)	1.587	0.878	0.071
Wet season farm access: walking (= 1)	1.997	0.979	0.04*
Perceived impact of canal block on harvest: increase (= 1)	5.987	157.340	0.967
Perceived impact of canal block on harvest: decrease (= 1)	− 4.797	1.304	0.000***
Perceived impact of canal block on access: no (= 1)	1.365	0.616	0.027*
Perceived impact of canal block on fire risk: no change (= 1)	− 2.347	0.707	0.000***
Existing canal block on farm: no (= 1)	− 1.170	0.692	0.091
Previously affected by peatland fire: no (= 1)	− 0.752	0.536	0.160

*** denotes *p* < 0.001, ** denotes *p* < 0.01, * denotes *p* < 0.05

### Canal block scenario 2

Of the 43 respondents who refused the 40-cm canal block, 58.1% were also unwilling to accept a canal block with a gate being built on their farm. Most (75%) of these respondents believed that this canal block would not work either (i.e. would have no effect on water level; 75%). As in the first scenario, these respondents stated that tidal changes in water level would stop the canal block from having any impact. The majority (60%) of respondents willing to accept this type of canal block stated that it would give them greater control over the water level (60%). See Fig. 3 and Table 5 for other reasons given by participants and example quotes.

**Table 5:** Example quotes from the second (with gate) canal block scenario

Willing to accept canal block	Category	Example quotes
Yes	Able to control water level	‘Because this would interrupt the farm less and you can control the water for irrigation’ (PL21) ‘Because there is a gate to control the water level’ (PL68)
	No impact on access	‘Because we can still use the canal for boat transport’ (PL20) ‘Can still access the farm by boat’ (M03)
	Improve irrigation	‘Because it will help irrigation’ (M40)
No	Negative farm impact	‘It will make the farm too wet’ (PL72) ‘Because it will still make the farm too wet to use the paths’ (PR01)
	Will note work	‘It will still be useless’ (M50) ‘It will have no effect’ (PL60)

**Fig. 5:**
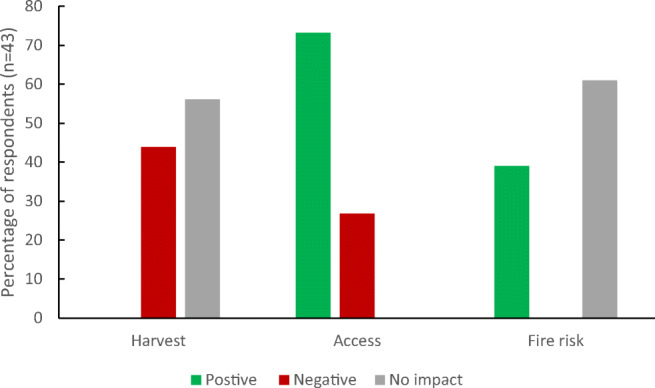
Perceived impacts of canal blocks with gates on yields, farm access and fire risk

We were unable to run a GLM for the second canal block scenario as the sample size for each predictor variable was too small. However, we can still draw insights from the quantitative and qualitative data. The majority of respondents to this scenario perceived that the canal block with a gate would have no impact on harvests, positive impacts on access and no impact on fire risk. However, there was a larger proportion of respondents perceiving negative impacts on yield in this subsample, compared with the entire sample (Figs. 4 and .5).

**Fig. 6:**
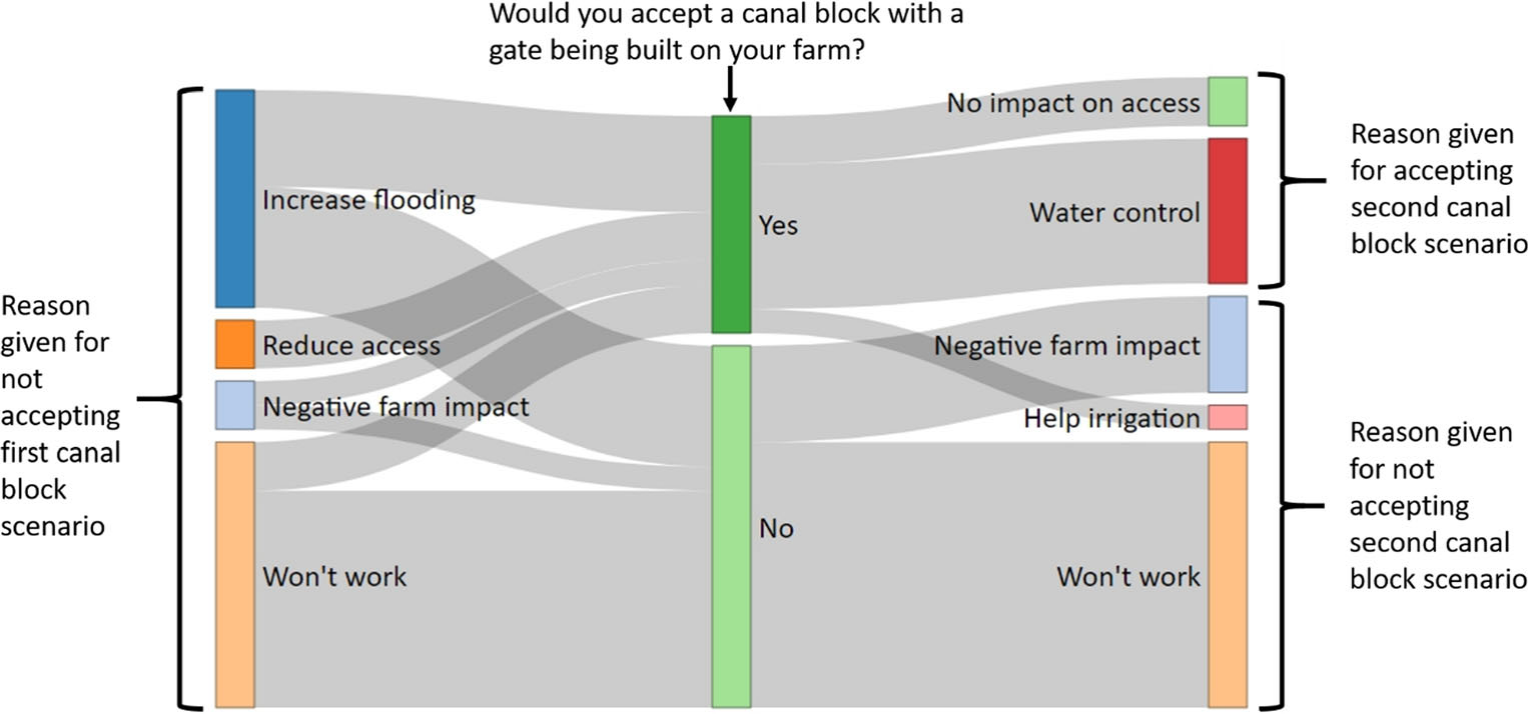
Sankey diagram showing reasons given for not accepting the first canal block scenario and reasons given for accepting or not accepting the second canal block scenario

Figure 6 shows the relational aspects of responses for not accepting the first canal block scenarios and their reasons for accepting or not accepting the second scenario. Of those respondents who were concerned about farm access by boat in the first scenario, all of them were willing to accept the canal block with a gate. However, the majority of respondents who stated that the first canal block would not work thought that the canal block with a gate would not work either. Respondents who perceived negative farm impacts and increased flooding were split on whether they thought the canal block with the gate would deal with these issues.

## Discussion

This research provides new evidence on the perceptions of smallholders towards peatland restoration efforts in the form of rewetting, targeting a much under-researched issue. Such studies are vital to informing the process adopted by restoration interventions in peatland areas globally. We found that the majority of smallholder farmers were willing to have canal blocks built on their farms; however, there was a range of perceptions about how the canal blocks may impact their farm access, yields and fire risk. In this section, we put our findings into the wider context of peatland restoration to outline how and when smallholders could be involved in peatland restoration given the findings from our study and how their perceptions could be utilised to inform restoration design.

### Mixed perceptions and mixed evidence

The majority of respondents were willing to have canal blocks built on their farms. This is a positive finding for BRG and peatland rewetting in Indonesia, as canal blocks can help to increase water table levels reducing the risks of subsidence, fires and reducing carbon emissions (Ritzema et al. 2014). There is also substantial evidence to suggest that environmental interventions are more likely to succeed when they have local support. Yet further research is needed to understand how large an area of peatland one canal block can help to rewet (Jaenicke et al. 2011; Yuliani and Erlina 2018). We nevertheless urge caution in assuming that there would be widespread acceptance of canal blocks by smallholder farmers in other locations in Indonesia, as this is a relatively small sample size, our respondents raised a number of concerns, and some of the reasons given for accepting canal blocks may not live up to expectations. We are also aware of the risk of acquiescence bias, where participants tend to agree with questions regardless of the connotations. Although we tried to alleviate this by giving explanations of the changes that each canal block would lead to, it may have led to inflated figures of respondents willing to accept canal blocks.

Respondents had mixed perceptions over whether canal blocks will affect yields, yet even within the scientific community, there is a lack of evidence to show the impact of raising water tables on yields of oil palm and other crops. A presidential decree in Indonesia stipulates that the water table in peatlands should not be more than 40 cm below the surface level, yet there appears to be little scientific evidence behind this decision (Page et al. 2009; Wardhana 2016; Dohong et al. 2018; Sabiham et al. 2018). Research has shown that water table levels in peatlands are highly variable and naturally range between 40 cm below and 100 cm above ground level (Wösten et al. 2008). Whilst another study suggested that raising the water level to 40 cm could reduce subsidence rates by 25–30% (Evans et al. 2019), other researchers argue that this level of drainage will still continue to degrade peatlands (Wijedasa et al. 2017; Sabiham et al. 2018). There is also limited evidence to show what impact raising water levels will have on oil palm yields. When the decree was announced, the Indonesian Palm Oil Association stated that it could lead to a 10% reduction in yield (Bell 2015), but empirical data are lacking. The small sub-sample of our respondents with existing canal blocks reported that there had been no noticeable impact since they had been installed. The majority of these respondents also told us that these canal blocks were still working. However, we would be cautious in over-interpreting this finding. Firstly, these canal blocks had all been installed relatively recently (with a median age of 2 years prior to data collection). Although there may have been immediate changes to water levels on farms, this may have not been enough time to have noticed changes in crop harvests, particularly with yearly variations depending on rainfall levels. Secondly, this represented the minority of our sample (36/181, 19.9%) and therefore is not large enough from which to draw wider conclusions. Thirdly, it is unusual to question authority in Indonesia. Although we explained that we were independent from the government, respondents may not have been willing to be open with us and to be seen as criticising government approaches. There have been some reports of canal blocks being sabotaged within the literature (Ritzema et al. 2014; Dohong et al. 2018), and anecdotally, we saw a number of blocks that did not seem to be functioning as they should. It is clear from our findings and the wider literature that better long-term data collection is needed to understand whether canal blocks are having an impact on yields. This may need to incorporate methodologies designed to investigate sensitive issues (St. John et al. 2010).

If there is a yield decline in response to rewetting, large plantation companies may be able to shift to non-peatland areas and find technological solutions. However, smallholder farmers will be affected most, with low access to capital for technological solutions, and few options to switch crops or move to a different area. Further research is urgently needed to understand what the impact of raising water tables will be on smallholder yields and to identify opportunities to share this knowledge with smallholder farmers, particularly as smallholders are already concerned about this aspect. It is possible that the private sector may have data on how water table impacts yields, and by engaging with these companies to explore their data, it could provide some answers, although farming methods will differ greatly between large-scale plantations and smallholders. The lack of information is nevertheless likely to be contributing to the mixed perceptions found in our research.

If raising the water table is likely to decrease yields, then there may be a need for compensation or a payment for ecosystem service (PES) scheme to ensure that the costs of restoration are not being borne by smallholder farmers, whilst benefits of restoration in biodiversity and carbon sequestration terms are shared out nationally and internationally. On the other hand, rewetting could in fact increase yields, due to oil palm requiring high water input, but may reduce overall profits due to difficulties in accessing farms and harvesting crops. Schaafsma et al. (2017) found that households in peatland areas in Kalimantan were willing to accept monetary compensation for switching from rubber and rice agriculture to tree planting, although many households were uncertain about whether they would receive payments. PES schemes have been used successfully in a range of countries and contexts where farmers are managing their land in a way that is beneficial for the environment but likely to reduce their yields or income, for example, via agri-environmental policies in the EU and USA (Baylis et al. 2008). However, careful implementation and design is needed to ensure that all households affected receive the compensation (e.g. Poudyal et al. 2016). This requires an emphasis to be placed on stakeholder participation and engagement in future restoration activities, as discussed below.

### Rewetting and restoration on the ground

Research, NGO and government publications on the process of restoration outline that different aspects, such as rewetting, revegetation and revitalisation of livelihoods should be implemented simultaneously (e.g. Dohong 2017; Graham et al. 2017; Dohong et al. 2018), although experts also emphasise that rewetting needs to take place before revegetation in order for the plants to grow successfully (Ward et al. 2020). In our research site, we found that only canal blocks were being implemented widely, with a few trial plots for livelihood projects and revegetation. Whilst this makes sense for revegetation, as discussed above, if there are any negative impacts to livelihoods from canal blocks, then the revitalisation aspect of BRGs approach needs to ensure that other viable livelihood options are offered alongside canal block building.

We found that the majority of smallholders who already had canal blocks on their farms felt that their opinions had not been listened to when these were built. Free prior informed consent (FPIC) is a key foundation to the BRG’s methods (Wardhana 2016; Dohong 2017), yet there may be barriers to its comprehensive implementation on the ground. Research on the use of FPIC in the forestry sector through programmes such as REDD+ has revealed ambiguities surrounding its interpretation and implementation, particularly in contexts with unclear property rights and complex governance systems (Mahanty and McDermott 2013). In a recent study of environmental management landscape approaches across Indonesia, experts cited a lack of transparency as the main barrier in achieving their project goals (Langston et al. 2019). The BRG has a deputy in charge of ‘Education, Information, Participation and Partnership’, and through this office, guidelines have been produced on engaging with villagers. However, these need to focus on ensuring that the communication lines can go both ways allowing knowledge exchange and for local people to raise their concerns and suggestions. Indonesia has a decentralised governance system meaning responsibilities need to be clear as to which institutions should handle which areas (both geographical and thematic). NGOs can play a supporting role in facilitating stakeholder engagement through capacity building, consensus building and trust building. However, it is also key to take the local context into account when establishing new partnerships, ensuring that NGO involvement does not undermine existing traditional power authorities or enable elite capture (Dyer et al. 2013; Ward et al. 2018a, 2018b, 2018c). To overcome potential issues and create solutions that are locally acceptable, it is crucial that all stakeholders are able to participate in environmental management decision making and that they are engaged from the very beginning (Stringer et al. 2017). Stakeholder participation can vary in timing and level of participation (Stringer et al. 2006; Reed et al. 2014; Orchard and Stringer 2016), and where local stakeholders are able to participate, interventions have been found to be more likely to succeed (de Vente et al. 2016; Sterling et al. 2017). However, participation must be meaningful and representative in order to be effective, ensuring that stakeholders are truly part of decision-making processes and all social groups are represented (Dyer et al. 2014; Ward et al. 2018a). Given our findings, participation could help to ensure that smallholders fully understand both the benefits and costs of installing canal blocks. This would enable smallholders to make an informed decision over whether canal blocks should be installed on their land, whilst opening up opportunities for dialogue so that their questions can be answered by project staff.

Participation could also provide an opportunity for local stakeholders to inform practitioners about local conditions, such as the tidal changes which many respondents mentioned as the reason they perceived the canal blocks would not work. This could allow practitioners and local stakeholders to come up with canal block designs which alleviate smallholders’ fears and explicitly discuss any potential trade-offs. Explanations from researchers or policy-makers of how the canal blocks work may help some farmers to change their perceptions; however, farmers will also have access to local knowledge which could contribute to a better design and planning for canal blocks, considering locally specific conditions (Raymond et al. 2010; Reed et al. 2014; Tschirhart et al. 2016). Knowledge co-production and exchange between researchers, local stakeholders and policy makers enables more effective knowledge creation, sharing and application in order to manage environmental issues, and increases local empowerment and ownership of projects (Dyer et al. 2014; Reed et al. 2014).

### Education and awareness raising

The most important factors in predicting whether farmers were willing to accept canal blocks were perceived impacts on harvest and fire, rather than household or socio-economic factors. For example, qualitative data showed that those who thought canal blocks would have a negative impact on harvests were concerned about having no control over the water level in their farms. This concern is pertinent given that there are issues with flooding in the wet season and drying out in dry season. The 40-cm canal blocks are specifically designed to ensure that the water is still able to drain to a certain extent, preventing flooding and also retaining water during the dry season (Suryadiputra et al. 2005; Dohong et al. 2018). Clearer explanations to smallholders regarding how canal blocks work may therefore be able to alleviate some of their concerns. In a review of community conservation interventions, Waylen et al. (2010) found that those including outreach and education were more likely to change attitudes than those that did not. Yet perceptions are often not rational or based on ‘objective data’, meaning that information campaigns aiming to improve knowledge will not necessarily lead to a change in attitudes (Bennett 2016). Therefore, it is key to implement explanations alongside opportunities for local stakeholders to participate in decision-making and knowledge sharing, as explained above. Addressing the challenges outlined in earlier sections regarding the lack of evidence to show exactly what the impacts of keeping water table depth at 40 cm will mean for agricultural (particularly oil palm) yields would also feed into this.

Respondents who perceived that canal blocks would decrease fire risk were more likely to accept a canal block being built on their farm. This suggests that discussions with smallholders around the risks of fire and how canal blocks will impact this may improve acceptability. However, there may be a trade-off between reduced fire risk and yield, and as stated above, further evidence is needed on the impact of canal blocks on crop yields. Additional research could also explore this trade-off further, to investigate what reduction in yield smallholders would consider acceptable for differing levels of fire risk reduction. Reducing peatland drainage in smallholder oil palm farms may not completely remove the risk of fire (particularly in El Niño years), and therefore, there is a need to be clear about this from the start, so that smallholders do not feel misled or that unrealistic expectations are set (Jefferson et al. 2020). There are many other fire management interventions currently being implemented across Indonesia, including new regulations, technical innovations, community fire monitoring and incentives for land management without fire (Chokkalingam et al. 2005; Carmenta et al. 2017; Jefferson et al. 2020). All of these fire management techniques vary in their effectiveness and acceptability (Carmenta et al. 2017). A cost-benefit analysis could be used to assess which combination(s) of methods for fire reduction offer the greatest cost-effectiveness in terms of economics, fire reduction and social acceptability.

Respondents who were concerned about farm access via boat in the first scenario were willing to have a canal block with a gate built on their farm. Qualitative data suggested that this was because it gave the farmers more control over the water level and because they could still use canals for boat travel. We were surprised to find that only 12% of our respondents relied on boats to access their farms, given that this was a concern raised by key stakeholder discussions and in the literature (Schaafsma et al. 2017; Graham et al. 2017). Other peatland areas may have much higher proportions of farmers reliant on canals to access their farms, and further research is needed to fully explore the impacts of canal blocks on farm access. This shows the importance of engaging with stakeholders before building the canal blocks, to understand which design type may be most appropriate. This approach would also allow a dialogue about the pros and cons of different canal blocks. Blocks with gates allow continued use of canals for boats, which is crucial in some areas, but inclusion of a gate needs more moving parts which may require greater maintenance and be more likely to break (Suryadiputra et al. 2005; Ritzema et al. 2014; Dohong et al. 2018). Another concern about blocks with gates is that the farmers have control over water levels and therefore may just leave the gates open preventing blocks from having any impact on water levels (particularly if they do not fully understand what the blocks are supposed to achieve). For these reasons, 40-cm blocks are likely to be the default rewetting strategy but, as discussed, may not be appropriate everywhere. Enabling local people to be part of the decision-making process may increase understanding about why different block types will be appropriate for different locations and the positives and negatives of each type.

We also found that some (25/181) respondents were not willing to have any kind of canal block on their farms, due to perceptions that they would have negative impacts on their farms, or would not work. Although this was a minority, it is still important to explore the reasons behind this. Qualitative data showed that this was due to beliefs that tidal changes were responsible for water level changes in the peatland meaning canal blocks would have little impact. As peatlands are naturally low-lying, it is possible that the water level is impacted by tidal changes. However, if canal blocks with the 40-cm spillway or gates are installed, then farmers will still have some control over water levels (Dohong 2017). We were unable to explore the influence of tidal changes in our research as all our villages were roughly equal distance from the coast, so further research is needed in this regard. As discussed above, knowledge exchange between smallholder farmers and technical experts designing canal blocks could provide opportunities to jointly create solutions (Reed et al. 2014; Stringer et al. 2017).

We did not find any differences in willingness to accept canal blocks between socio-economic factors, such as income, livelihood or age, with the exception of ethnicity, discussed further below. Our sample included a good range of incomes and ages, with no obvious outliers, so it seems that these are not important factors in determining acceptance of canal blocks. As the majority of our sample relied on oil palm for their income, this is maybe not surprising: if farmers perceive that canal blocks will have no impact on their harvests, as we found, then this will be equally important for all incomes and ages. For those farmers who perceived that the canal block would negatively impact their farms, the reasons that they gave would be equally problematic regardless of income or age. We also found that whilst one of our villages had a lower acceptance rate than the other two, yet there were no significant differences in socio-economic factors (e.g. income, livelihood, ethnicity) between the villages. Informal discussions suggested that this difference might have been caused by perceived negative impacts of canal blocks in a plantation near to village 2, and from our anecdotal observations, these farms already appeared to be much wetter than those in the other villages. This emphasises how perceptions can differ within similar groups based on past experiences (Bennett 2016).

In this research, we found that respondents of Sumatran ethnicity were more likely to agree to canal blocks compared with those migrants from Java or Sulawesi. Indonesia has a history of transmigration, both spontaneous and government-organised programmes, where people from more populated islands are encouraged to move to areas with lower populations (van Lottum and Marks 2012; Yulmardi et al. 2018). Schaafsma et al. (2017) found a similar difference when investigating the levels of compensation that local communities would need, in order to participate in a peatland tree-planting scheme. They showed that indigenous households were more likely to support canal blocking than transmigrant households. The majority of transmigrant households in our study area were from Java, which does not contain any peatlands. In Kalimantan (Indonesian Borneo), transmigrant farmers have tried to use farming methods learnt from their previous experiences on mineral soil, leading to low yields and land degradation (Uda et al. 2018). In the case of the government-organised transmigration, peatlands were often drained by large-scale projects, such as the Mega Rice project in Kalimantan (Page et al. 2009; Lilleskov et al. 2019). Other research has suggested that in cases where transmigrant communities have been moved to areas where they struggle to farm successfully, they are less likely to support local or national land management interventions (van Beukering et al. 2008; Yulmardi et al. 2018). Again, knowledge exchange between new or transmigrant villages and indigenous villages could help to share more successful and sustainable methods of farming used by farmers who have been living in peatland areas for many generations (Tschirhart et al. 2016). Nevertheless, such farming methods that are considered sustainable in small areas may not continue to be sustainable if population sizes start to grow. Another potential solution for farmers living in peatland areas is to switch to aquaculture, given that peatlands naturally contain many fish species, or paludiculture. Paludiculture focuses on species which naturally grow in peatland (Dohong 2017; Gunawan 2018; Dohong et al. 2018); however, further research is needed to explore the economic value of these species and the market viability of such a switch.

## Conclusion

Tropical peatland restoration is globally important for health, environmental and economic reasons. However, in areas where peatland is currently being used for agriculture, restoration activities, including rewetting, will have an impact on smallholder farmers. Our findings provide the first published research insights into local stakeholders’ perceptions of peatland rewetting initiatives in Indonesia and add to the scientific literature showing the importance of understanding local stakeholders’ perceptions of environmental management interventions. We found that the majority of smallholder farmers would accept a canal block being built on their farm; however, this varied depending on how they perceived canal blocks to impact their yields and change fire risk and whether they are able to access their farms via alternative transport to going by boat. More research is needed to understand the impact of raising water levels on smallholders’ crops. Understanding farmers’ perceptions is central if the government is to meet its targets for peatland restoration, and this requires stakeholder engagement from the outset of restoration efforts. Such early engagement can help to deliver a more even distribution of the costs and benefits of restoration between farmers and other stakeholders in the restoration process.

## Supplementary information

ESM 1 (DOCX 94 kb)
