# Supplementary material for: Smallholder perceptions of land restoration activities: rewetting tropical peatland oil palm areas in Sumatra, Indonesia
Source: Reg Environ Change. 2020 Dec 19;21(1):1. doi: 10.1007/s10113-020-01737-z (PMC7749744; doi:10.1007/s10113-020-01737-z)
Supplement: Supplementary file 1 — (DOCX 94 kb) [file 10113_2020_1737_MOESM1_ESM.docx]

**Supplementary online material**

Online Resource 1: Numerical variables used in the model to analyse the factors influencing whether smallholder farmers would accept a canal block being built on their farm

| Variables | Mean | Standard Deviation |
| --- | --- | --- |
| Age (years) | 42.2 | 12.0 |
| Household size (number of people) | 4.2 | 1.3 |
| Income (million rupiah per month) | 2.7 | 7.7 |
| Number of income generating activities | 1.6 | 0.59 |

Online Resource 2: Categorical variables used in the model to analyse the factors influencing whether smallholder farmers would accept a canal block being built on their farm

| Variables | Summary (n=181) |
| --- | --- |
| Perceived impact on yield | Increase: 48  Decrease: 21  No change: 111 |
| Perceived impact on farm access | Yes: 27  No: 152 |
| Perceived impact on fire risk | Increase risk: 0  Decrease risk: 116  No change: 62 |
| Village | Village 1: 80  Village 2: 41  Village 3: 60 |
| Ethnicity (region respondent was born in) | Born in village: 61  Other area in Sumatra: 48  Java: 65  Sulawesi: 7 |
| Education | None: 16  Elementary: 106  High School: 37  Vocational: 17  University: 5 |
| Main income activity | Oil palm: 143  Areca nut: 20  Coconut: 4  Other: 14 |
| Previously received a government subsidy | Yes: 25  No: 156 |
| Farm access in wet season | Walking: 47  Motorbike: 106  Boat: 24 |
| Current canal use | Yes: 83  No: 97 |
| Previously heard of canal blocks | Yes: 94  No: 87 |
| Canal block on farm currently | Yes: 36  No: 145 |
| Canal block near farm | Yes: 22  No: 158 |
| Previous fire experience | Yes: 50  No: 128 |

**Online Resource 3: Questionnaire**

**Introduction**

We are a team of researchers from the UK and Indonesia working together to understand the impact (good or bad) of restoring peatland on local communities, biodiversity, water and carbon emissions.

One part of our research aims to understand what people think about different peatland management approaches. For this we are asking smallholder oil palm farmers some questions about your lives, farms and thoughts about canal blocking. These questions should take about 45 minutes. There are no right or wrong answers, we are just interested in your opinions. Your answers will be used in our research but your name will not be included. Taking part is completely voluntary and you can stop the interview at any point. We are independent researchers and have neutral views about peatland management so are just interested in learning from you. We cannot promise that we will improve your lives or change existing systems.

**Verbal consent**

We would like to ask your consent, i.e. whether you willingly accept to talk to us. If you choose to take part, you can still stop us at any time or refrain from answering any questions you are not comfortable with.

Would you like to continue with the questionnaire?

| Date |  |
| --- | --- |
| Research assistant |  |
| Village |  |
| Start time |  |
| End time |  |

**Socio-economic information**

1. Were you born in this village? Yes No

If no

- 1. Where are you originally from?

| Other part of Sumatra |  |
| --- | --- |
| Java |  |
| Sulawesi |  |
| Kalimantan |  |
| Other____________________ |  |

- 1. How long have you lived here (in years)?____________________
  2. Why did you move here?_________________________

1. Age______________
2. Education level

| None |  |
| --- | --- |
| Elementary |  |
| High school |  |
| Vocational |  |
| College/Master’s |  |

1. How many people live in your household?____________________
2. Annual income (including farm and off-farm) Rp:_______________________________________
3. What activities do you do to earn money?
   1. Which activities do you earn the most money from?

| Activity | Rank (1 =most income earnt, 5= least income earnt) |
| --- | --- |
|  |  |
|  |  |
|  |  |
|  |  |
|  |  |

1. Have you ever received any form of support from the government (e.g. transmigrant subsidy, food subsidy, farmers community support, other?)

| Yes | Type_______________  Amount Rp:__________  Year________________ |
| --- | --- |
| No |  |

**Farm information**

1. What crops do you grow on your farm? (Tick all that apply)
   1. What area do these crops grow on (check unit)
   2. What year did you plant these crops?
   3. Why did you choose to grow these crops?
   4. Which crops do you earn the most money from?

|  |  | Area covered (check unit) | Year planted | Reason for farming | Which do you earn the most (1=most money earnt) |
| --- | --- | --- | --- | --- | --- |
| Oil palm |  |  |  |  |  |
| Areca |  |  |  |  |  |
| Coconut |  |  |  |  |  |
| Banana |  |  |  |  |  |
| Other___________ |  |  |  |  |  |

1. What are the most important things when you are choosing which crops to farm?

1.___________________________________________________________________

2.___________________________________________________________________

3.___________________________________________________________________

1. How do you access your farm in the dry season?

Boat Walking Motorbike Car Other

1. How do you access your farm in the wet season?

Boat Walking Motorbike Car Other

**Canal block scenarios**

In this section we would like to understand your preferences for different peatland management approaches. We have designed this as a game, in which you will be asked to consider different scenarios.

Even though we frame it as a game, we ask you to answer as you would in a real situation

**Scenario 1**

Imagine that an organisation wants to build a canal block on your farm. This canal block will keep the water level at 40cm minimum all of the time. This means that the ground will remain wet in the dry season. Some water will be able to drain in the rainy season, but some water will remain..

1. Would you accept this canal block being built on your farm? Yes No
2. Why/why not?______________________________________________________________________________

___________________________________________________________________________________________

1. How do you think the canal block would affect your oil palm harvest?___________________________________
2. Would it affect access to your farm? Yes No

____________________________________________________________________________________________

1. Do you think the canal block would change the risk of fire on your farm?

| Increased risk of fire |  |
| --- | --- |
| Decreased risk of fire |  |
| No change |  |

1. Has thinking about impacts on crop harvest, transport and fire risk changed your mind on whether you would accept the canal block being built on your farm? Yes No
   1. If yes, why did you change your mind?_____________________________________

___________________________________________________________________

(if no, continue to scenario 2. If yes, continue to question 37)

**Scenario 2**

Imagine that an organisation wants to build a canal block on your farm. This canal block will have a gate so that you can control the water level on your farm. The land will remain wet in the dry season. Some water will be able to drain in the rainy season, but the ground will remain wet.

Because there is a gate, you will still be able to use a boat to travel on the canal.

1. Would you accept this canal block being built on your farm? Yes No
2. Why/why not?______________________________________________________________________________

___________________________________________________________________________________________

1. How do you think the canal block would affect your oil palm harvest?___________________________________
2. Would it affect access to your farm? Yes No

____________________________________________________________________________________________

1. Do you think the canal block would change the risk of fire on your farm?

| Increased risk of fire |  |
| --- | --- |
| Decreased risk of fire |  |
| No change |  |

1. Has thinking about impacts on crop harvest, transport and fire risk changed your mind on whether you would accept the canal block being built on your farm? Yes No
   1. If yes, why did you change your mind?_____________________________________

___________________________________________________________________

**Canal blocking and fire experience**

1. Do you use the canals in the peatland?
2. If yes, what for?______________________________________________________________
3. Had you heard of canal blocking before this questionnaire? Yes No

If yes

1. Has there been a canal block on your farm? Yes No

(If yes continue. If no, go to question 38b)

- - 1. When was it built?__________________
    2. What type of canal block is it? Full block 40cm block block with gate
    3. Why was it built? _____________________________________________________________________________________________________________
    4. Who decided to build it?_____________________________________
    5. Do you feel your views were listened to before the canal block was built?

Yes No

Why/why not?_________________________________________________

- - 1. Were you involved in building it? Yes No
    2. Is the canal block still on your farm now? Yes No
    3. What impact has the canal block had on you and your household? (tick all that apply)

| Lower crop yield |  |
| --- | --- |
| Difficulty travelling to farm |  |
| Difficulty managing farm |  |
| Difficulty transporting crops |  |
| Changing crops grown |  |
| Other______________________ |  |

If no

1. Has there been a canal block anywhere near your farm? Yes No
   1. How far from your farm is it?_______________
   2. Is the canal block still there now? Yes No
   3. Does the canal block affect you accessing your farm? Ya Tidak
2. Have there been any fires in or near your farm in the last 10 years? Yes No
3. If yes, did these cause any problems? What were these problems? (prompts if needed: health, property/crop damage, schooling)______________________________________________________________________________________________________________________________________________
4. Do you have any questions or comments?______________________________________

Online Resource 4: Main income generating livelihood activities and their differing uses of canals
